# mRNA vaccines encoding computationally optimized hemagglutinin elicit protective antibodies against future antigenically drifted H1N1 and H3N2 influenza viruses isolated between 2018-2020

**DOI:** 10.3389/fimmu.2024.1334670

**Published:** 2024-03-12

**Authors:** James D. Allen, Ted M. Ross

**Affiliations:** ^1^ Center for Vaccines and Immunology, University of Georgia, Athens, GA, United States; ^2^ Department of Infectious Diseases, University of Georgia, Athens, GA, United States; ^3^ Florida Research and Innovation Center, Cleveland Clinic, Port Saint Lucie, FL, United States; ^4^ Department of Infection Biology, Lerner Research Institute, Cleveland Clinic, Cleveland, OH, United States

**Keywords:** influenza, vaccine, mRNA, universal, hemagglutinin, H1N1, H3N2

## Abstract

**Background:**

The implementation of mRNA vaccines against COVID-19 has successfully validated the safety and efficacy of the platform, while at the same time revealing the potential for their applications against other infectious diseases. Traditional seasonal influenza vaccines often induce strain specific antibody responses that offer limited protection against antigenically drifted viruses, leading to reduced vaccine efficacy. Modern advances in viral surveillance and sequencing have led to the development of in-silico methodologies for generating computationally optimized broadly reactive antigens (COBRAs) to improve seasonal influenza vaccines.

**Methods:**

In this study, immunologically naïve mice were intramuscularly vaccinated with mRNA encoding H1 and H3 COBRA hemagglutinins (HA) or wild-type (WT) influenza HAs encapsulated in lipid nanoparticles (LNPs).

**Results:**

Mice vaccinated with H1 and H3 COBRA HA-encoding mRNA vaccines generated robust neutralizing serum antibody responses against more antigenically distinct contemporary and future drifted H1N1 and H3N2 influenza strains than those vaccinated with WT H1 and H3 HA-encoding mRNA vaccines. The H1 and H3 COBRA HA-encoding mRNA vaccines also prevented influenza illness, including severe disease in the mouse model against H1N1 and H3N2 viruses.

**Conclusions:**

This study highlights the potential benefits of combining universal influenza antigen design technology with modern vaccine delivery platforms and exhibits how these vaccines can be advantageous over traditional WT vaccine antigens at eliciting superior protective antibody responses against a broader number of influenza virus isolates.

## Introduction

Influenza viruses can cause serious respiratory illnesses in humans which are linked to 2.9 – 6.5x10^5^ mortalities globally every year (1–5). Influenza viruses are also responsible for acute infections that cause many people to miss valuable hours at work or school (6, 7). Seasonal vaccines are available for influenza and annual vaccination is recommended for all people over 6 months old by the U.S. Centers for Disease Control and Prevention (CDC) and the Advisory Committee on Immunization Practices (ACIP) (8, 9). However, influenza viruses are antigenically variable and therefore, they frequently escape host immune pressures through a process called antigenic drift (2, 4, 10, 11). During this process, the virus gradually introduces amino acid substitutions in immunodominant epitopes of its surface glycoproteins hemagglutinin (HA) and neuraminidase (NA) (5, 10, 12, 13). As a result, seasonal influenza vaccines must be updated frequently to remain antigenically similar to the viruses actively in circulation (14–16). Seasonal vaccines in the U.S. are typically comprised of inactivated wild-type (WT) viruses selected by the World Health Organization (WHO) (9, 17, 18). The viruses included in the seasonal vaccine each year are chosen based on predictions of which viral clades will be dominant or emerging in the upcoming year, but because of the time it takes to manufacture large quantities of the vaccine, this selection occurs ~6-months prior to the release of the vaccines (14, 19). Over that period the viruses in circulation can antigenically drift from the selected strain, leading to an immunologic mismatch between the vaccine and the viruses in circulation (13, 14, 17, 20). This was exemplified in the 2018-2019 influenza season, in which a vaccine mismatch resulted in a low protective efficacy of ~41% against H1N1 viruses, and ~9% against H3N2 viruses (19, 21, 22). Thus, a vaccine that can be manufactured quickly, updated rapidly, and provides protection against a diverse population of antigenically drifted influenza viruses is needed (2, 5, 20, 23).

Production time to manufacture influenza vaccines can fluctuate depending on the delivery platform, such as inactivated virus, live attenuated virus, recombinant subunit, peptide, virus-like particle, mRNA, and DNA vaccines (24–26). Among those, mRNA vaccines are advantageous as they can be manufactured rapidly, generate protective immune responses, use no infectious material in their production, and offer advantages over other platforms, such as DNA, because there is no risk of incorporating into the host genome (24, 25, 27). The production speed of mRNA vaccines was exemplified in 2021, when vaccines for SARS-CoV-2 were advanced from receipt of the sequence to phase 1 clinical trials in ~2 months (17, 20). mRNA encoding vaccine antigens also generate protective antibodies and robust antigen specific cellular immune responses in vaccinated animals (18, 28, 29). As a result, the use of the mRNA platform to deliver the next generation of influenza vaccines has gained a significant amount of interest in recent years (24, 26, 27, 30).

In addition to increasing production speeds, influenza virus vaccines also need to be updated rapidly to be antigenically matched to the viruses in circulation (17, 20, 26). Therefore, it is necessary to select antigenically similar wild-type strains closer to the start of flu season or use more broadly reactive vaccine antigens that induce protection against immunologically drifted isolates of influenza (17, 18, 26, 27). Recent advances in viral surveillance and sequencing have led to the development of various *in-silico* methodologies for generating broadly reactive vaccine antigens (31–33). Previously, our group has reported on a method of generating computationally optimized broadly reactive antigens (COBRAs) for influenza virus vaccine development (34–38). This methodology utilizes influenza virus HA protein sequence information from currently circulating strains to generate broadly reactive vaccine antigen sequences. These HA antigens are effective in murine, ferret, and non-human primate models at increasing immunologic breadth against panels of historical and future drifted influenza isolates when compared to wild-type influenza vaccine antigens (39–45). In addition, these vaccine antigens can be updated in real time to antigenically represent actively circulating viruses and broaden their protective immune responses (35, 36). Thus, incorporating the COBRA technology with a vaccine delivery platform like mRNA, that can be produced rapidly and generate protective immune responses, may be highly advantageous for the field of influenza virus vaccinology.

Overall, this was the first time that mRNA encoding COBRA HA antigens were synthesized and used to vaccinate immunologically naïve mice to determine their effectiveness at eliciting broadly protective antibody responses against diverse panels of H1N1 and H3N2 vaccine strains from the last decade. These responses were superior to those elicited by wild-type mRNA expressing HA antigens at inhibiting viral binding to host cell surface receptors and preventing viral infection. Thus, demonstrating the potential benefits of using *in-silico* designed influenza mRNA vaccine antigens to elicit more broadly protective immune responses over traditional wild-type vaccine antigens.

## Materials and methods

### Antigen sequences

Previously generated COBRA H1 and H3 HA sequences were generated using full length, 566 amino acid, wild-type influenza virus HA protein sequences from isolates in circulation from 2014-2018. The Y2 H1 COBRA HA sequence was designed using sequences from human H1N1 viruses in circulation from May 1, 2014 – September 30, 2016, and the NG2 H3 COBRA HA sequence was designed from human H3N2 HA sequences in circulation from May 1, 2016 – April 30, 2018 (42, 46). All sequences used in the design process were obtained from the Global Initiative on Sharing Avian Influenza Data (GISAID) EpiFlu online database. The sequences were then used to produce consensus-based HA sequences using the *in-silico* COBRA design algorithms as described previously (36). In addition to the COBRA Y2 and NG2 HAs, the sequences of the A/California/07/2009 H1N1 HA (EPI_ISL_159428), and the A/Kansas/14/2017 H3N2 HA (EPI_ISL_403059) were also downloaded from GISAID.

### mRNA and lipid nanoparticles

Sequence optimized mRNAs encoding either COBRA Y2 H1 HA, COBRA NG2 H3 HA, A/California/07/2009 H1 HA, or A/Kansas/14/2017 H3 HA were synthesized as described previously (47). After synthesis, the mRNAs were purified, sterile filtered, and moved to long term storage at -20°C. The mRNA was then encapsulated in lipid nanoparticles (LNP) through a previously described modified ethanol-drop nanoprecipitation procedure (48).

### Viruses and recombinant HA proteins

All H1N1 and H3N2 influenza viral isolates were acquired through the U.S. Centers for Disease Control (CDC), BEI Resources, the Influenza Reagents Resource (IRR), or provided by Virapur (San Diego, CA, USA). The influenza viruses were passaged in embryonated chicken eggs following protocols provided by the World Health Organization (WHO) (49). The hemagglutination content of the H3N2 influenza virus lots were determined using 0.75% guinea pig red blood cells in the presence of 20nM Oseltamivir. Once quantified, the virus stocks were made into single use 1mL aliquots and stored at -80°C for future use. Similarly, the hemagglutination content of the H1N1 viruses were quantified using 0.8% turkey red blood cells, made into single use 1mL aliquots, and stored at -80°C until use.

The seven historical H3N2 vaccine strain viruses used in this study were: A/Hong Kong/4801/2014 (HK/14) egg passage 11 (EP11) (clade 3c.2a), A/Singapore/IFNIMH-16-0019/2016 (Sg/16) EP3 (clade 3c.2a1), A/Kansas/14/2017 (Kan/17) EP1 (clade 3c.3a), A/Switzerland/8060/2017 (Switz/17) EP1 (clade 3c.2a2), A/South Australia/34/2019 (SA/19) EP1 (clade 3c2.a1b/131K), A/Hong Kong/2671/2019 (HK/19) EP1 (clade 3c.2a1b.131F), A/Tasmania/503/2020 (Tas/20) EP1 (clade 3c.2a1b.2a.1). The four historical H1N1 vaccine strain viruses were: A/California/07/2009 (Cal/09) EP4 (clade pdm09), A/Brisbane/02/2018 (Bris/18) EP1 (clade 6B.1A.1), A/Guangdong-Maonan/SWL1536/2019 (Guang/19) EP1 (clade 6B.1A.5A.2), A/Victoria/2570/2019 (Vic/19) EP1 (clade 6B.1A.5A.1).

### Vaccination and viral infection of mice

Female DBA/2J mice (n=110, 6-8 weeks old) were purchased from The Jackson Laboratory (Bar Harbor, ME, USA). Mice were housed in microisolator units and provided unlimited access to food and water for the entire study. Mice were cared for under the USDA guidelines for laboratory animals. The procedures performed on the mice were approved by the University of Georgia Institutional Animal Care and Use Committee (IACUC) (no. A2021-06-016-Y3-A6). At the beginning of the study the mice were randomly divided into 10 groups (n=11 mice/group), and vaccinated intramuscularly with 50μL of a solution containing 1μg of mRNA vaccines encoding the HA proteins of either the H1 COBRA Y2, H1 A/California/07/2009 (EPI_ISL_159428, EP3), H3 COBRA NG2 or H3 A/Kansas/14/2017 (EPI_ISL_403059, MDCK-SP2) in phosphate buffered saline (PBS) (Thermo Fisher, Waltham, MA, USA), or 50μL of PBS alone as a placebo control vaccine (24, 30, 50). The vaccines were administered as either a monovalent (1μg) or bivalent (H1 + H3) (1μg each) formulation intramuscularly into the hamstring of the hind leg of the mice on the first day of the study and again on day 28. Blood samples were obtained from the facial vein of the animals on day 14 and day 42 post initial vaccination. Whole blood samples were centrifuged at 2,500 rpm for 10 minutes to obtain serum. The clarified serum layer was then extracted from each individual sample and stored at -20 ± 5°C.

On day 56 post initial vaccination, 55 of the mice (n= mice/group) were infected with 50μL of a human A/California/07/2009 H1N1 influenza isolate that has been passaged in eggs (EP4) at a dose of 5x10^4^ PFU/50μL. Also on day 56 post initial vaccination, another set of 55 mice (n=11 mice/group) were challenged with 50μL of a human A/Kansas/14/2017 H3N2 influenza isolate that has been passaged in eggs (EP1) at a dose of 1.55x10^7^ PFU/50μL. Mice were observed for 14 consecutive days after infection for weight loss and clinical signs of infection. A humane 25% weight loss cut off was observed for all mice, post infection, as previously established by the IACUC committee. On the third day of the viral challenge, 3 mice from each group were humanely euthanized, and their lungs were removed, snap frozen, and stored at -80°C. Following day 14 of the influenza virus challenge, all mice were humanely euthanized using IACUC approved methods (no. A2021 06-016-Y2-A6).

### Hemagglutination inhibition assay

The hemagglutination inhibition assay was utilized to quantify anti-HA antibodies that can prevent the binding of influenza viruses to red blood cells (RBCs). The protocol used for this study was reproduced from the WHO laboratory influenza surveillance manual (49). Turkey RBCs were used for the assays with A(H1N1) influenza viruses, and guinea pig RBCs were used in assays with A(H3N2) influenza viruses. Guinea pig RBCs are commonly used to characterize modern influenza A(H3N2) viruses isolated since 2005, which preferentially bind to alpha (2, 6) linked sialic acids on the surface of host cells (51, 52). Before beginning the assay, nonspecific inhibitors present in the sera were inactivated by treating samples with receptor-destroying enzyme (RDE) (Denka, Seiken, Co., Japan) according to the manufacturer’s instructions. In brief, three volumes of reconstituted RDE was mixed with one volume of sera and was allowed to incubated overnight at 37°C. After 16 hours, the samples were heat treated by incubating them at 56°C for 30 minutes, and then six volumes of PBS were mixed with each sample.

For HAI assays run with influenza A(H1N1) viruses, 50μL of treated sera was added to the first column of 96-well V-bottom plates (Thermo Fisher, Waltham, MA, USA). and then 25μL was transferred across the plate in a series of two-fold serial dilutions into the remaining wells which contained 25μL of PBS. Next, 25μL of influenza virus, adjusted to 8 hemagglutination units (HAU)/50μL in PBS, was added to each well. The plates were then allowed to incubate at 22°C for 20 minutes. After incubation, 50μL of a solution containing 0.8% turkey RBCs (Lampire Biologicals, Pipersville, PA, USA) in PBS was added to each well, and the plates were mixed by gentle agitation and incubated for another 30 minutes at 22°C. Before the assay, the RBCs were washed 2x with PBS, stored at 4°C, and used within the next 24 hours. After incubation with RBCs the plates were tilted to observe hemagglutination. The HAI titer was then quantified by taking the reciprocal of the dilution of the last well that contained non-agglutinated RBCs. Serum from previously performed mouse infections with matched influenza A(H1N1) viruses, at day 14 post infection, was also included on each plate to confirm assay consistency.

For HAI assays run with influenza A(H3N2) viruses, 50μL of treated sera was added to the first column of 96-well V-bottom plates and then 25μL was transferred across the plate in a series of two-fold serial dilutions into the remaining wells which contained 25μL of PBS. Next, 25μL of influenza virus, adjusted to 8 hemagglutination units (HAU)/50μL in PBS, supplemented with 20nM Oseltamivir carboxylate (Aobious, Gloucester, MA, USA) was added to the plate. The plates were then allowed to incubate for 30 minutes. After incubation, 50μL of a solution containing 0.75% guinea pig RBCs (Lampire Biologicals, Pipersville, PA, USA) diluted in PBS was added to each well, and the plates were mixed by gentle agitation and incubated for 1 hour at room temperature. Before the assay, the RBCs were washed twice with PBS and stored at 4°C, before being used in the next 24 hours in the assay. After incubation with RBCs the plates were tilted to observe hemagglutination. The HAI titer was determined by taking the reciprocal dilution of the last well that contained non-agglutinated RBCs. Serum from previously performed mouse infections with matched influenza A(H3N2) viruses, at day 14 post infection, was also included on each plate to confirm assay consistency.

Prior to the study, all mice were bled, and were determined to be devoid of pre-existing HAI reactive antibodies to human influenza viruses. For this study, a positive HAI reaction (HAI+), or “sero-protection” is defined as an HAI titer ≥ 1:40, as per the WHO and European Committee for Medicinal Products guidelines for evaluating influenza vaccines (53).

### Focal reduction assay

The Focus Reduction Assay (FRA) was used for assessing influenza neutralizing antibodies present in mouse serum from vaccinated animals. The protocol used for the FRA was written by the WHO collaborating Centre in London, U.K. and adapted by the U.S. Centers for Disease Control and Prevention (CKC) (Thomas Rowe, personal communication). Madin-Darby Canine Kidney SIAT1 (MDCK-SIAT1) cells (Sigma, St. Louis, MO, USA) were seeded into 96-well flat-bottom plates (Thermo Fisher, Waltham, MA, USA) at a density of 2.5-3.5x10^5^ cell/mL (100μL/well), one day prior to the assay. Cells were cultured in Dulbecco’s Modified Eagle Medium (DMEM) (Thermo Fisher, Waltham, MA, USA), containing 5% heat-inactivated fetal bovine serum (Thermo Fisher, Waltham, MA, USA) and 1% Penicillin/Streptomycin (100U/mL Penicillin, 100μg/mL Streptomycin solution) (Thermo Fisher, Waltham, MA, USA). The following day, when cells reach ~95% confluency, cell monolayers were washed two times with sterile 0.01M PBS pH 7.2 (Thermo Fisher, Waltham, MA, USA). Next, serum samples were pooled for each group of vaccinated mice, and serially diluted 2-fold, starting at a dilution of 1:20, in virus growth medium (VGM-T) containing DMEM that is supplemented with 1μg/mL of TPCK-treated trypsin, 1% Penicillin/Streptomycin (100U/mL Penicillin, 100μg/mL Streptomycin solution), and 0.1% BSA (Thermo Fisher, Waltham, MA, USA). The PBS was then removed from the 96-well plates, and 50μL samples from each serum dilution was added to the plate. Next, 50μL of VGM-T containing influenza virus (600 FFU/50μL) was added to the wells of each plate, or VGM-T alone was added to negative control wells (54, 55). The influenza A(H1N1) viruses used for this assay were: A/California/07/2009 EP4, A/Brisbane/02/2018 EP1, and A/Guangdong-Maonan/SWL/1536/2019 EP1. The influenza A(H3N2) viruses used for this assay were: A/Kansas/14/2017 EP1, A/Hong Kong/2671/2019 EP1, and A/South Australia/34/2019 EP1.

After the addition of virus to the plates, they were incubated at 37°C + 5% CO_2_ for 2h. After incubation, they were overlaid with 100μL of equal volumes of 1.2% Avicel (Type RC581; FMC Health and Nutrition, Philadelphia, PA, USA) in 2x Modified Eagle Medium (MEM) (Thermo Fisher, Waltham, MA, USA) containing 0.1% BSA, 1μg/mL TPCK-treated trypsin, and 1% Penicillin/Streptomycin and incubated at 37°C + 5% CO_2_ for 18-22 hours. The overlay was then removed from each well, and the plates were washed with PBS. The plates were then fixed with 100μL of 4% phosphate buffered formalin (Thermo Fisher, Waltham, MA, USA) in each well for 30 minutes at 4°C. After being fixed, the plates were washed once with PBS, and a 100μL of permeabilization buffer (0.5% Triton-X-100 (Thermo Fisher, Waltham, MA, USA) in PBS/glycine) is added to each well and allowed to incubate for 20 minutes at room temperature. The permeabilization buffer is then removed and the plates were washed 3x with wash buffer (PBST) (PBS, 0.1% Tween-20), and allowed for 1 hour with 50μL per well of a primary antibody solution containing mouse anti-influenza a nucleoprotein monoclonal antibody (IRR, Manassas, VA, USA, FR1217, 1mg/mL), diluted 1:2000 in ELISA buffer (PBS, 0.1% Tween-80, 10% heat inactivated horse serum). After incubation, the primary antibody solution was discarded, and the plates were washed 3x with PBST. Then, 50μL of secondary antibody solution (goat anti-mouse peroxidase labeled IgG (Seracare, Inc., Milford, MA, USA, KPL 474-1802, 1mg/mL) diluted 1:2000 in ELISA buffer) is added to each well for 1 hour at room temperature. Next the plates were washed 3x with PBST, and influenza infected cells were stained with TrueBlue peroxidase substrate (SeraCare, Inc., Milford, MA, USA) supplemented with 0.03% H_2_O_2_. Plates were incubated with substrate for 10 min, then the plated were rinsed 5x with tap water. After air drying overnight, the foci were counted using a CTL BioSpot Analyzer with ImmunoCapture 6.4.87 software (CTL, Shaker Heights, OH, USA). The neutralization titer was determined by taking the reciprocal of the highest dilution of the well that corresponded to a 50% foci reduction compared to the average of the virus control wells.

For consistency, all plates were run in duplicate, the virus control wells needed to have counts between 200 and 1600 foci, and the negative control wells must not have any foci. Additionally, wells containing murine reference sera, must have similar FFU counts across the plates. Each assay plate also contains serum from humans that received commercial influenza vaccines to assess overall assay consistency (56). The percent infected cells reported in the assay was calculated by dividing the of FFU counts in each experimental well by the average FFU counts of the positive control wells.

### Influenza viral plaque assay

Madin-Darby Canine Kidney (MDCK) epithelial cells (Sigma, St. Louis, MO, USA) were cultured and added to each well of a six-well plate (Thermo Fisher, Waltham, MA, USA) at a concentration of 1x10^6^ cells/well, 18-24h before the assay. The next day, lung tissues were weighed and homogenized in 1mL of DMEM supplemented with 1% Penicillin/Streptomycin (DMEM + P/S) (Thermo Fisher, Waltham, MA, USA). Lung homogenates were then pelleted at 2,000 rpm for 5 min, and the supernatants were harvested. Supernatants were then diluted in 10-fold serial dilutions in DMEM + P/S. When MDCK cells in six-well plates reached ~90-95% confluency, the plates were rinsed 2x with DMEM + P/S, and then 100μL of each dilution of the lung homogenate supernatant in duplicate. The plates were then allowed to incubate for 1h. During the hour the plates were agitated every 15mins. Following incubation, the media was removed, and cells were rinsed 2x with DMEM + P/S. Next, the DMEM + P/S was removed, and a 50:50 v/v mixture of 2X MEM and 1.6% agarose (Thermo Fisher, Waltham, MA, USA) supplemented with 1μg/mL of TPCK Trypsin (Thermo Fisher, Waltham, MA, USA) was added to each well. The solution is then allowed to solidify at room temperature, and the plates are incubated at 37°C + 5% CO_2_ for ~72 h. After 72 h, the solidified agarose gels were manually removed, and the cells were fixed for 10 min with a 10% buffered formalin solution (Thermo Fisher, Waltham, MA, USA). The formalin was then discarded, and the plates were stained with a 1% crystal violet solution (Thermo Fisher, Waltham, MA, USA) for 10 min. The crystal violet was then removed, and the plates washed 5x with fresh water. The plates were then allowed to dry for 24 hours, after which the plaques are counted and reported as the number of plaques in the reciprocal of each dilution. The lung vial titers are then presented as plaque forming units per gram of lung tissue (PFU/g) by comparing the number of viral plaques to the original weight of the lung tissue from each animal.

### Statistical analysis

Data is presented as an absolute mean value +/- the standard error of the mean (SEM). A nonparametric one-way analysis of variance (ANOVA) was used to determine the statistical differences amongst groups using GraphPad Prism software (GraphPad, San Diego, CA, USA). For this study, a p value <0.05 was defined as statistically significant (*=p <0.05, **=p <0.01, ***=p <0.001, ****=p <0.0001). In other cases, the statistical significance was determined using unpaired Mann-Whitney t-Tests on GraphPad Prism software. A p value <0.05 was defined as statistically significant (*=p <0.05, **=p <0.01, ***=p <0.001, ****=p <0.0001).

## Results

### COBRA HA mRNA vaccination induces broader sero-protective HAI antibody responses than wild-type HA mRNA vaccination

**Figure 1:**
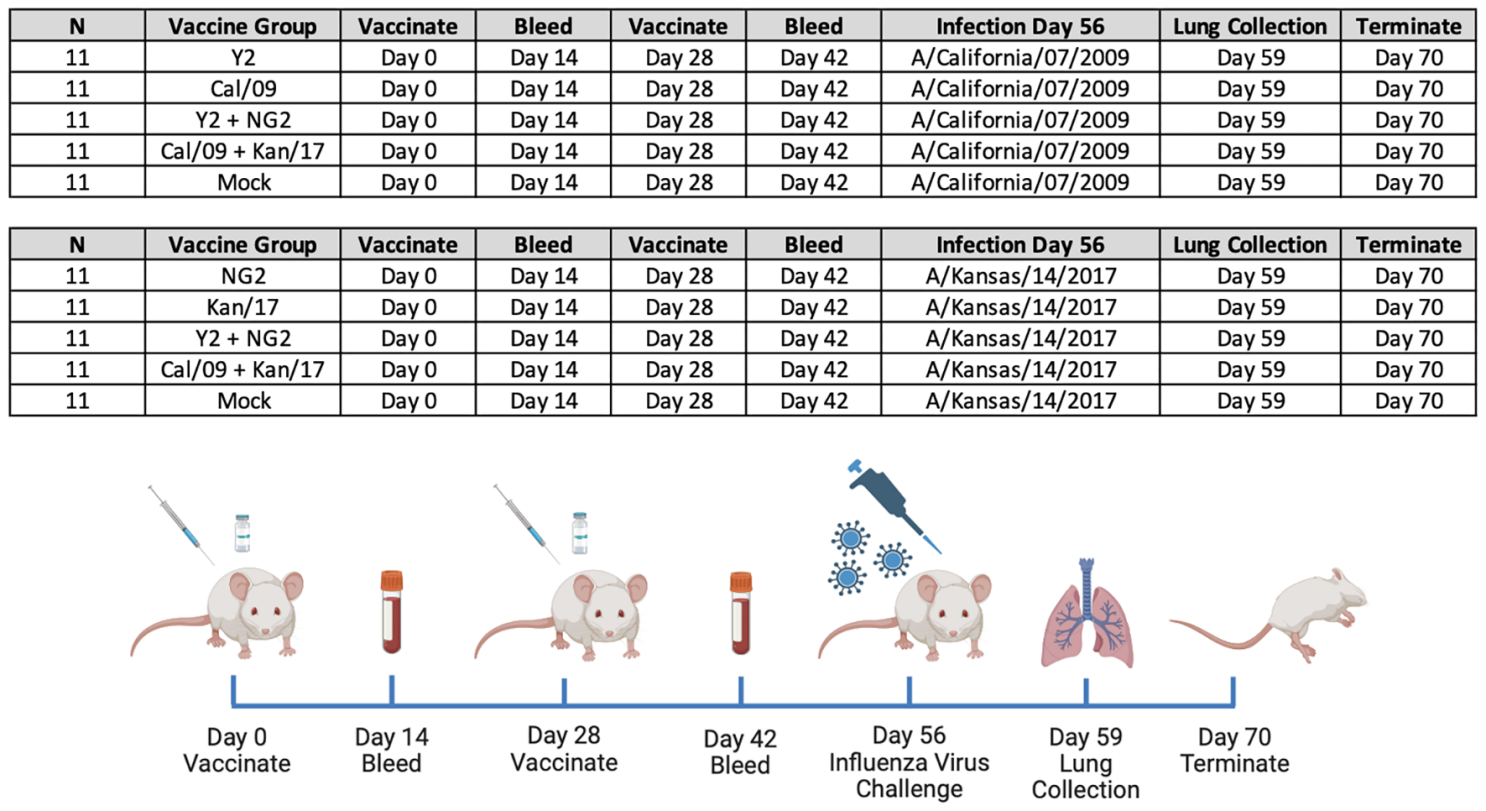
Groups and Study Timeline. 110 immunologically naïve DBA/2J mice split into 10 groups (n=11/group) and primed on day 0 with 1μg of mRNA LNPs expressing either monovalent or bivalent formulations of COBRA or wild-type HA. On day 14 animals were bled to collect serum for serological assays. Two weeks later, on day 28, the mice in each group were boosted with a homologous mRNA LNP vaccine, and 14 days later, on day 42 blood was collected. On day 56, 55 of the animals vaccinated with mRNA LNPs expressing H1 HA antigens were infected with A/California/07/2009 H1N1 influenza virus, and 55 of the animals vaccinated with mRNA LNPs expressing H3 HA antigens were infected with A/Kansas/14/2017 H3N2 influenza virus. Following the infection, body weight and clinical symptoms were tracked in each animal for 14 consecutive days. Lungs were collected from 3 animals in each group three days following infection, on day 59 of the study. On day 70 all animals in the study were humanely euthanized and the study was terminated.

**Figure 2:**
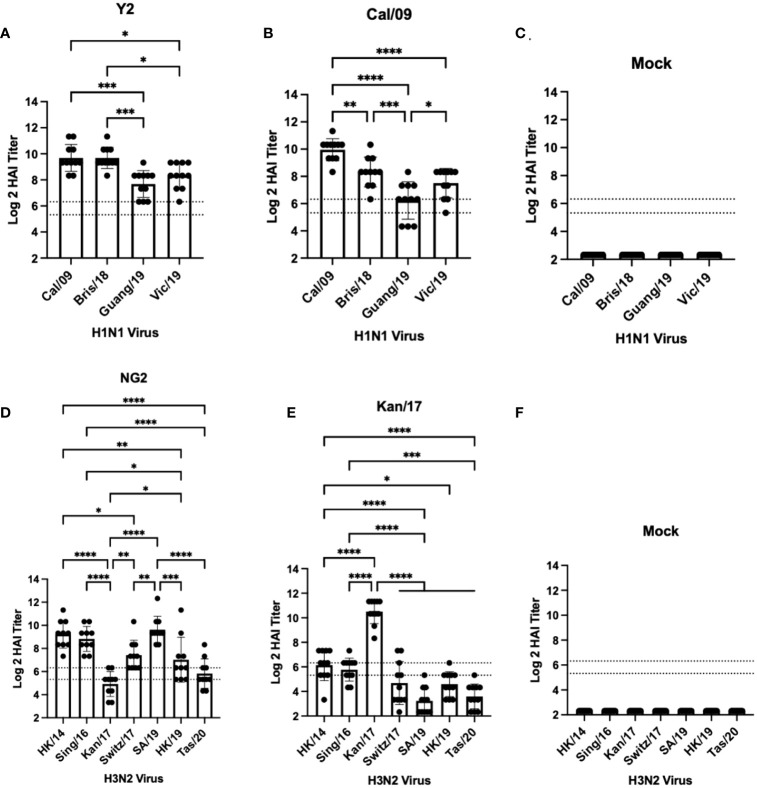
Day 42 HAI Response: Monovalent Vaccines. Serum collected from 66 DBA/2J mice (n=11/group) on day 42 were assessed for HAI reactive antibodies against 4 H1N1 isolates from 2009-2019 **(A–C)** or 7 historical H3N2 vaccine strains isolated from 2014-2020 **(D–F)** (X-axis). Log2 HAI titers are reported for each animal (Y-axis). The lower dotted line depicts an HAI titer of 1:40. The upper dotted line depicts an HAI titer of 1:80. Mice were vaccinated two times with 1μg of mRNA LNPs encoding either COBRA Y2 HA **(A)**, WT Cal/09 HA **(B)**, mock control vaccines containing PBS **(C, F)**, COBRA NG2 HA **(D)**, or WT Kan/17 HA **(E)**. Significance was determined using nonparametric one-way ANOVA. A p value <0.05 was defined as statistically significant (*=p <0.05, **=p <0.01, ***=p <0.001, ****=p <0.0001).

**Figure 3:**
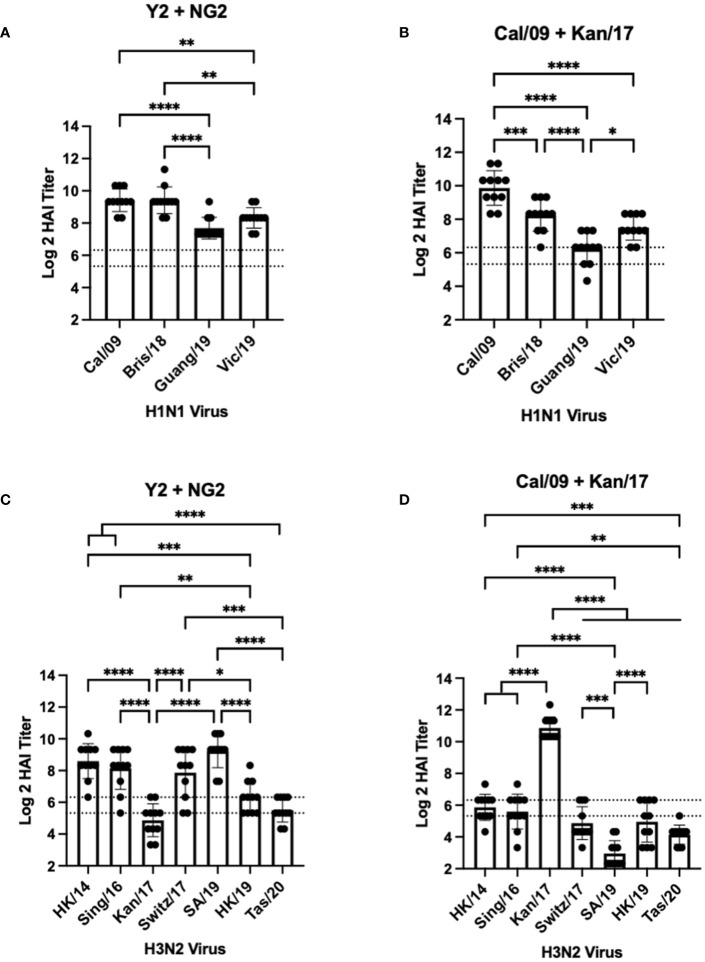
Day 42 HAI Response: Bivalent Vaccines. Serum collected from 44 DBA/2J mice (n=11/group) on day 42 were assessed for HAI reactive antibodies against 4 H1N1 isolates from 2009-2019 **(A, B)** or 7 H3N2 isolates from 2014-2020 **(C, D)** (X-axis). Log2 HAI titers are reported for each animal (Y-axis). The lower dotted line depicts an HAI titer of 1:40. The upper dotted line depicts an HAI titer of 1:80. Mice were vaccinated two times with 1μg of mRNA LNPs encoding bivalent mixtures of either COBRA Y2 + NG2 HA **(A, C)** or WT Cal/09 + WT Kan/17 HA **(B, D)**. Significance was determined using nonparametric one-way ANOVA. A p value <0.05 was defined as statistically significant (*=p <0.05, **=p <0.01, ***=p <0.001, ****=p <0.0001).

DBA/2J mice (n=11/group) were vaccinated in a prime-boost regimen with either monovalent or bivalent formulations of COBRA HA-encoding or wild-type HA-encoding mRNA vaccines. Vaccinated mice were infected with either H1N1 or H3N2 influenza viruses 28 days after the booster vaccination (**Figure 1**). Sera were collected 14 days post-boost and were assessed for HAI reactive antibodies against a panel of 4 H1N1 isolates from 2009-2019, and 7 H3N2 isolates from 2014-2020 (**Figures 2**, **3**). All mice immunized with the COBRA Y2 H1 HA-encoding mRNA vaccine had seroprotective antibody titers (≥1:80) against the H1N1 viruses from 2009-2019. The highest antibody titers were directed against the Cal/09 and Bris/18 viruses and these responses were significantly higher (p<0.001) than the antibody responses against Guang/19 and Vic/19 (**Figure 2A**). Mice immunized with Cal/09 H1 HA-encoding mRNA vaccine on average generated seroprotective antibodies against the H1N1 strains from 2009-2019, however 3 out of the 14 animals (21.4%) had titers ≤1:40 against the Guang/19 isolate. The highest antibody responses in these animals were directed against the Cal/09 virus and these titers were significantly higher than those elicited against the viruses from 2018-2019 (**Figure 2B**). Mock vaccinated animals had no detectable antibody titers against the H1N1 viruses in the panel (**Figure 2C**). Animals immunized with the NG2 COBRA H3 HA-encoding mRNA vaccine generated seroprotective antibodies against 6 of the 7 H3N2 strains isolated from 2014-2020. The highest HAI reactive antibody titers were directed against the HK/14 and SA/19 strains and these responses were significantly higher than those against the Kan/17, Switz/17, HK/19, and Tas/20 viruses. The lowest HAI reactive antibody responses elicited by these vaccines were directed against the Kan/17 virus, however 7 of the 11 vaccinated animals (63.6%) had HAI activity (≥1:40) against this strain (**Figure 2D**). Mice immunized with Kan/17 H3 HA-encoding mRNA vaccine had an average seroprotective HAI titer (≥1:40) against 3 of the 7 H3N2 vaccine strains, all of which were in circulation from 2014-2017. The highest HAI activity in these animals was directed against the homologously matched Kan/17 virus and this response was significantly higher than those elicited against any of the other H3N2 strains isolated between 2014-2020. On average, Kan/17 H3 HA-encoding mRNA vaccinated animals had little or no seroprotective HAI titers against the future drifted isolates from 2019-2020. Sera from these animals had the lowest HAI activity against the SA/19 (**Figure 2E**). There was no detectable HAI activity in polyclonal sera collected from mock vaccinated animals against the H3N2 viruses (**Figure 2F**).

Mice vaccinated with a bivalent (H1+H3) HA-encoding mRNA vaccine were assessed for HAI reactive antibodies 14 days after the booster vaccination against the same H1N1 and H3N2 viral panels as the mice vaccinated with monovalent vaccine formulations (**Figure 3**). Similar to the mice vaccinated with Y2, each mouse immunized with a bivalent COBRA Y2 + NG2 HA-encoding mRNA vaccine elicited seroprotective HAI antibody titers (≥1:80) against all the H1N1 viruses isolated from 2009-2019. The highest antibody titers from these animals were elicited against the Cal/09 and Bris/18 viruses and these responses were significantly higher than those against the Guang/19 and Vic/19 viruses (**Figure 3A**). Mice vaccinated with a bivalent Cal/09 H1 + Kan/17 H3 HA-encoding mRNA vaccine had HAI titers that were on average >1:40 against the H1N1 viruses from 2009-2019. However, not all mice in this group had sera with detectable HAI activity. The highest HAI titers were detected against the homologously matched Cal/09 virus. These HAI titers were significantly higher than titers against any of the other viruses in the panel. These mice had the lowest HAI activity against the Guang/19 virus and this response was significantly lower than those directed against the other viruses isolated between 2018 and 2019 (**Figure 3B**). Serum from the mice immunized with a bivalent Y2 + NG2 COBRA HA-encoding mRNA vaccine was also assessed for HAI activity against H3N2 viruses isolated between 2014-2020. On average, these animals had HAI activity against 6 of the 7 strains in the H3N2 panel. The highest HAI titers were elicited against the SA/19, and this was significantly higher than the titers against the Kan/17, HK/19, and Tas/20 viruses. The lowest HAI activity was directed against the Kan/17 isolate, but 6 of the 11 animals had HAI activity against this virus (**Figure 3C**). Mice vaccinated with the bivalent Cal/09 + Kan/17 HA-encoding mRNA vaccine, on average, had antibodies with HAI+ activity against 3 of the 7 strains isolated from 2014-2020 and all 3 of those viruses were isolated between 2014-2017. The highest HAI activity was elicited against Kan/17 virus, and the titers were significantly higher than those generated against the other H3N2 viruses. Similar to monovalent Kan/17 HA vaccination, serum samples from mice vaccinated with a bivalent Cal/09 + Kan/17 HA-encoding mRNA vaccine had the lowest HAI titers against the SA/19 strain with HAI titers ≥1:40 in all the 11 animals (**Figure 3D**).

### COBRA HA mRNA vaccines elicited significantly higher HAI titers against future drifted H1N1 and H3N2 isolates than mRNA vaccines expressing WT HA proteins

**Figure 4:**
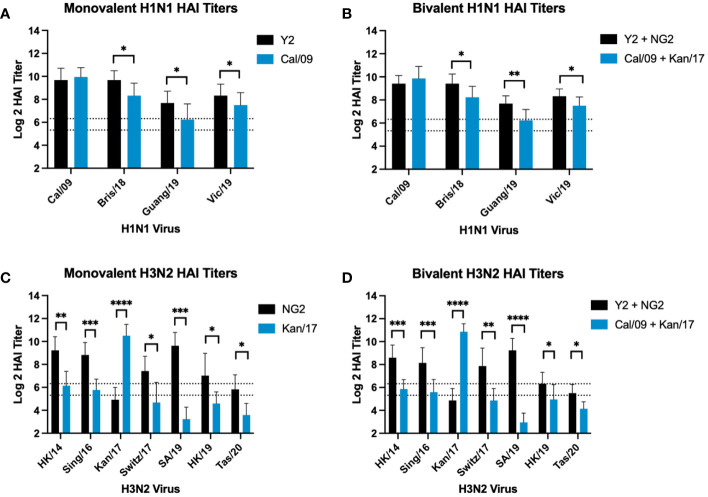
Comparison of Day 42 HAI Response: Monovalent & Bivalent Vaccines. Serum collected on day 42 were assessed for HAI reactive antibodies against 4 H1N1 isolates from 2009-2019 **(A, B)** or 7 H3N2 isolates from 2014-2020 **(C, D)** (X-axis). Log2 HAI titers are reported for each animal (Y-axis). The lower dotted line depicts an HAI titer of 1:40. The upper dotted line depicts an HAI titer of 1:80. Mice were vaccinated two times with 1μg of mRNA LNPs encoding either monovalent formulations of COBRA Y2 HA (black) or WT Cal/09 HA (blue) **(A)**, bivalent mixtures of either COBRA Y2 + NG2 HA (black) or WT Cal/09 + WT Kan/17 HA (blue) **(B, D)**, or monovalent formulations of COBRA NG2 HA (black) or WT Kan/17 HA (blue) **(C)**. Significance was determined using unpaired Mann-Whitney t Tests. A p value <0.05 was defined as statistically significant (*=p <0.05, **=p <0.01, ***=p <0.001, ****=p <0.0001).

The same HAI antibody titers from mice vaccinated with monovalent or bivalent formulations of COBRA HA-encoding or WT H1 and H3 HA-encoding mRNA vaccines were compared head-to-head across after the boost against a panel of H1N1 and H3N2 viruses from 2009-2020 (**Figure 4**). In both formulations, animals vaccinated with COBRA Y2 HA-encoding mRNA vaccine had significantly higher HAI titers than animals vaccinated with the WT Cal/09 mRNA against future drifted H1N1 vaccine strain isolates from 2018-2019 (**Figures 4A, B**). The WT Kan/17 HA-encoding mRNA induced significantly higher HAI titers than the COBRA NG2 HA-encoding mRNA vaccine against the Kan/17 H3N2 isolate in monovalent and bivalent formulations. However, against the 6 other strains in the H3N2 panel, the COBRA NG2 HA-encoding mRNA vaccinated animals had significantly higher HAI titers than animals vaccinated with the WT Kan/17 mRNA (**Figures 4C, D**). No statistical differences, p>0.05, were observed in the HAI reactive antibody titers elicited by each antigen in either monovalent or bivalent formulations.

### COBRA HA mRNA vaccines generated more robust neutralizing antibody responses against future drifted H1N1 and H3N2 isolates compared to WT HA mRNA vaccines

**Figure 5:**
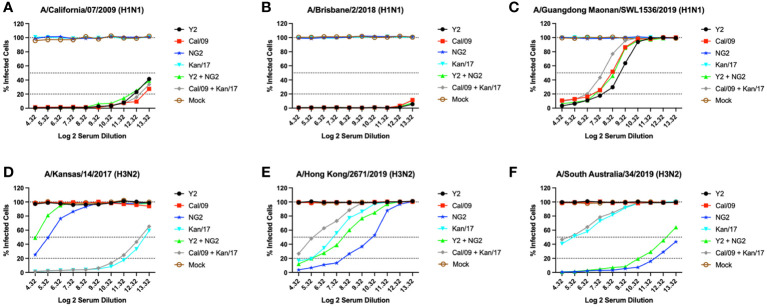
Day 42 H1N1 and H3N2 Neutralizing Antibody Titers. Serum from each group of DBA/2J mice was pooled (N=11/group) and tested for neutralizing antibody content against 3 H1N1 viruses, A/California/07/2009 **(A)**, A/Brisbane/02/2018 **(B)**, A/Guangdong-Maonan/SWL1536/2019 **(C)**, and 3 H3N2 viruses, A/Kansas/14/2017 **(D)**, A/Hong Kong/2671/2019 **(E)**, and A/South Australia/34/2019 **(F)**. Serum was diluted 2-fold down the plate and are represented as Log2 dilutions (X-axis). The lower dotted line illustrates a 80% neutralization of viral infection (Neut_80_), the middle-dotted line depicts a 50% neutralization (Neut_50_), and the upper most dotted line illustrates no neutralization.

Serum was collected from each group of mice at day 42 and was tested *in-vitro* for the ability to neutralize live virus in a focal reduction assay (FRA) against a panel of H1N1 and H3N2 influenza viruses (**Figure 5**). Mice that received vaccines containing either COBRA Y2 HA-encoding or WT Cal/09 HA-encoding mRNAs had high neutralizing antibody titers against the Cal/09 H1N1 virus. Mice immunized with formulations containing the Cal/09 HA-encoding mRNA vaccine had ~1.2-fold higher Neut_80_ (80% neutralization) titers than animals immunized with the COBRA Y2 HA-encoding mRNA vaccine against the Cal/09 virus (**Figure 5A**). All the animals vaccinated with formulations containing any of the H1 HA-encoding mRNAs, regardless of antigen, had serum neutralizing antibody titers >80% at all the serum dilutions against the Bris/18 H1N1 virus, with similar results from both COBRA HA-encoding and WT HA-encoding vaccinated animals (**Figure 5B**). Against the Guang/19 H1N1 virus, animals vaccinated with monovalent formulations of COBRA Y2 HA-encoding mRNA elicited serum neutralizing antibody titers that were ~1.4-fold higher than mice vaccinated with monovalent formulations of Cal/09 HA or bivalent formulations of Y2 + NG2 HA-encoding mRNAs. The Y2 vaccinated mice also had ~3-fold higher Neut_50_ titers than mice vaccinated with bivalent formulations of Cal/09 + Kan/17 HA-encoding mRNA vaccines. Animals that received the WT Cal/09 HA mRNA monovalent vaccine had ~1.56-fold higher Neut_50_ titers than the mice vaccinated with a bivalent WT formulation of Cal/09 + Kan/17 HAs (**Figure 5C**). Animals immunized with a monovalent H3 HA mRNA vaccine had no neutralizing antibodies that prevented infection by the Cal/09, Bris/18 or Guang/19 H1N1 viruses (**Figures 5A–C**).

Mice immunized with the WT Kan/17 HA-encoding mRNA vaccine had Neut_50_ titers that were ~16-fold higher than animals vaccinated with COBRA NG2 HA mRNA against the Kan/17 H3N2 virus, while the COBRA NG2 HA-encoding mRNA vaccinated mice had antibodies capable of neutralizing 50% of the Kan/17 viral infections at a serum dilution of ~1:40 (**Figure 5D**). Against the HK/19 virus, the animals vaccinated with COBRA NG2 HA-encoding mRNA had the highest serum neutralizing antibody titers and these titers were ~6.3-fold higher compared to the titers induced by mRNA formulations with the WT Kan/17 HA. The monovalent COBRA NG2 HA-encoding mRNA induced ~4.7-fold higher Neut_50_ titers than the titers induced in animals vaccinated with the bivalent Y2 + NG2 COBRA HA mRNAs. The WT Kan/17 HA-encoding mRNA had ~3-fold higher neutralizing antibody titers than those vaccinated with a bivalent formulation of WT Cal/09 + Kan/17 HA mRNAs, and their serum prevented 50% of the HK/19 infections at a dilution of ~1:120 (**Figure 5E**). Sera from mice vaccinated with COBRA NG2 HA mRNA formulation had Neut_50_ titers ~16-fold higher against the SA/19 virus compared to animals vaccinated with WT Kan/17 HA mRNA formulation. Monovalent formulation of COBRA NG2 HA mRNA induced ~1.38-fold higher neutralizing antibody titers than the bivalent formulation of Y2 + NG2 HA-encoding mRNAs. Mice vaccinated with either formulation of WT mRNAs encoding Kan/17 HA had similar levels of SA/19 neutralizing antibodies that prevented 50% infection at a dilution of ~1:40 (**Figure 5F**). Animals immunized with a monovalent H1 mRNA vaccine had no neutralizing antibodies that prevented infection caused by the Kan/17, HK/19, or SA/19 H3N2 viruses (**Figures 5D–F**).

### COBRA HA mRNA vaccines protected animals from a lethal A/California/07/2009 H1N1 infection and prevented viral replication in the host lungs

**Figure 6:**
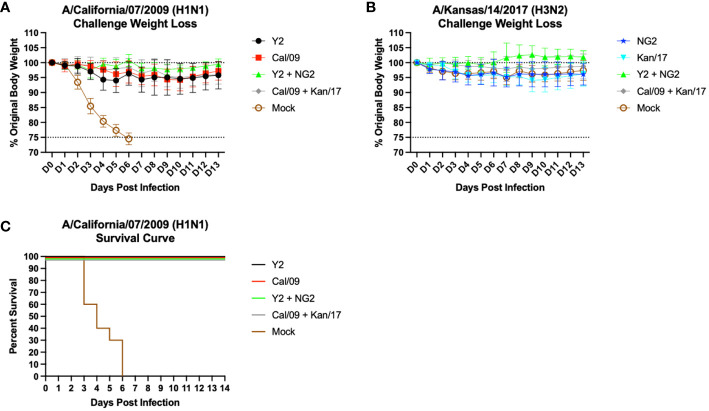
Day 56 Viral Challenge Weight Loss and Survival Curves. 55 DBA/2J mice (n=11/group) were infected on day 56 post initial vaccination with 5x10^4^ PFU/50μL of A/California/07/2009 H1N1 virus, and their weight loss was tracked for 14 consecutive days following viral infection **(A)**. Another set of 55 DBA/2J mice (n=11/group) were also infected on day 56 post initial vaccination with 1.55x10^7^ PFU/50μL of A/Kansas/14/2017 H3N2 virus, and their weight loss was tracked for 14 consecutive days following viral infection **(B)**. The lower dotted line depicts the humane endpoint (25% weight loss), and the upper dotted line depicts the % weight of the mice at the start of the infection (100%). Survival curves were also generated for the mice challenged with the A/California/07/2009 H1N1 virus **(C)**. Percent survival is for each group is reported (Y-axis) for each day following infection (X-axis).

Following the second vaccination, the mice were challenged with antigenically distinct seasonal influenza vaccine strains. Half of the mice were infected with a 10xLD_50_ dose of A/California/07/2009 H1N1 virus, 5x10^4^ PFU/50μL, to assess the protective efficacy of the mRNA vaccines (**Figures 6A, C**). The body weight loss of these animals was tracked for 14 days following infection. Animals immunized with an H1 HA mRNA vaccine, Y2 or Cal/09, lost on average 5-10% body weight, which peaked on day 5 post-infection. By day 6, most of the H1 HA mRNA vaccinated mice began gaining weight that was maintained for the duration of the 14 days. Mice vaccinated with mock vaccines lost ~25% body weight by day 6 and were humanely euthanized (**Figure 6A**). The H1 mRNA vaccinated animals survived the H1N1 viral challenge, while none of the mock animals survived past day 6 post infection (**Figure 6C**). The other half of the mice were challenged with 1.55x10^7^ PFU/50μL of A/Kansas/14/2017 H3N2 virus. This viral infection resulted in ~5-10% body weight loss in all groups, including the mock vaccinated animals. This weight loss peaked around day 9 post-infection, and some of the animals in the Y2 + NG2 group gained weight during the challenge when compared to their starting weight (**Figure 6B**). These results were expected since H3N2 influenza viruses isolated from humans typically do not cause severe disease in most murine models and, in this study, all mice survived the H3N2 viral challenge.

**Figure 7:**
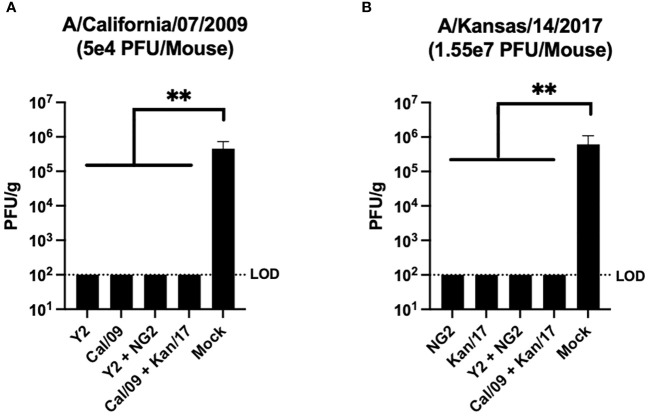
Day 59 Lung Viral Titers. Lungs collected from 3 mice per group on day 59 (3 post infection) were assessed to determine the viral content of the respiratory tissue. For this analysis, mice were separated into those infected with 5x10^4^ PFU/50μL of A/California/07/2009 H1N1 virus **(A)**, and those infected with 1.55x10^7^ PFU/50μL of A/Kansas/14/2017 H3N2 virus **(B)**. Vaccine groups are listed on the X-axis, and plaque forming units per gram (PFU/g) of lung tissue is depicted on the Y-axis. The dotted line was inserted to represent the limit of detection of the assay. A nonparametric one-way ANOVA. A p value <0.05 was defined as statistically significant (**=p <0.01).

Viral plaque assays were performed on lung samples collected from 3 mice in each group at day 3 post infection to assess the ability of the vaccines to prevent viral replication in host respiratory tissues (**Figure 7**). Mock vaccinated animals infected with the Cal/09 H1N1 virus had viral lung titers ~5x10^5^ PFU/g at day 3 post infection. This was significantly higher than any of the animals that received mRNA formulations expressing the COBRA Y2 HA or the homologously matched Cal/09 H1 HA, which had no detectable viral lung titers at day 3 post-infection (**Figure 7A**). Similar to the H1N1 influenza virus infected animals, the other set of mock vaccinated animals challenged with the Kan/17 H3N2 influenza virus also had viral lung titers ~5x10^5^ PFU/g at day 3 post infection. This was significantly higher than any of the animals that received COBRA NG2 HA-encoding mRNA vaccine or the homologously matched Kan/17 H3 HA mRNA vaccine, which had no detectable virus present in their lungs at day 3 post infection (**Figure 7B**).

## Discussion

In recent years, the development and commercialization of mRNA vaccines has revolutionized the field of vaccinology. Large quantities of mRNA vaccines can be produced in relatively short time frames compared to traditional methods of generating influenza virus vaccines in embryonated chicken eggs (17, 24, 26, 27). This is highly advantageous when designing vaccines against pathogens that undergo constant antigenic drift, like influenza viruses, where the ever-changing viral landscape can lead to reduced vaccine efficacy, if the antigens included in the vaccine are not closely related to circulating strains (17, 33, 57, 58). Additionally, mRNA vaccine platforms can be combined with an *in-silico* antigen design methodology for producing broadly reactive antigens, like COBRA, which utilizes sequence information from influenza surveillance centers to generate antigens that provide immune protection against a broader spectrum of viruses than traditional wild-type vaccine antigens (18, 59). This study demonstrates that the combination of the mRNA vaccines and COBRA technologies allow for the generation of immunologically effective, broadly reactive influenza vaccine candidates. Based on the nature of these platforms, vaccine candidates can be rapidly updated, and quickly mass produced to respond to the viruses currently circulating in the wild. Thus, reducing the likelihood of vaccine mismatch, while at the same time increasing antigenic breadth within a season of influenza circulation when compared to traditional wild-type vaccine antigens.

In this study, the Y2 H1 COBRA mRNA vaccine elicited similar HAI and neutralizing antibody titers as the Cal/09 mRNA vaccine against the A/California/07/2009 virus. Upon challenge with the Cal/09 H1N1 virus, the Y2 vaccine also prevented viral replication in the lungs of vaccinated animals and protected them against weight loss and mortality. Typically, in unvaccinated mice this virus is one of the most pathogenic H1N1 viruses isolated since the 2009 H1N1 pandemic, so it was very encouraging to observe that the Y2 COBRA vaccine could provide protection against this virus at a level that was comparable to the Cal/09 mRNA vaccine. Many modern H1N1 viruses are not as pathogenic in the mouse model, and thus the HAI and neutralization assays performed in this study were used as surrogates to assess the theoretical protection provided by these vaccines against future drifted H1N1 isolates from 2018-2019. In these assays, the Y2 COBRA mRNA vaccine induced higher HAI and neutralization antibody titers than the Cal/09 mRNA vaccine against future drifted H1N1 influenza viruses from 2019 (**Figures 4**, **5**). This is likely due to the sequence similarity of Y2 to these future drifted viruses. The Y2 and Cal/09 HA proteins differ in sequence by 14 amino acids, and these HA proteins share ~97.53% structural identity to one another, however, Cal/09 and the future drifted Guang/19 H1 HA differ by 23 amino acids, while Y2 and Guang/19 differ by 10 amino acids (60). Some of these amino acid differences between Y2, Cal/09, and Guang/19 occur in known antigenic sites of the H1 HA protein. H1N1 influenza viruses typically evade receptor binding site targeting monoclonal antibodies by introducing amino acid substitutions in antigenic site Sb, and in this region the Cal/09 HA has a 201S, Y2 has a 201T, and Guang/19 has a 201I (61). Serine and threonine are both hydroxylic amino acids, which are polar, hydrophilic, and uncharged at neutral pH, therefore it is unlikely that the observed immunogenic differences between Y2 and Cal/09 are being driven by the variance at site 201 (62, 63). This also occurs in antigenic site Ca1 at position 220, where the Cal/09 HA protein contains a serine, and the Y2 and Guang/19 HA contain a threonine. There are also differences in antigenic site Sa at position 180, where the Cal/09 HA protein contains a lysine, while Y2 and Guang/19 possess a glutamine. Lysine is positively charged at neutral pH, while glutamine is a non-charged, polar amino acid at normal physiological conditions (63). Therefore, the structural and electrostatic differences in these two amino acids may be a driving force in the observed immunological differences between the Cal/09 and Y2 HA. However, specific site directed mutagenesis experiments would need to be conducted at sites 180, 201, and 220 to determine if each of these differences, or a combination thereof, play a role in generating a stronger HAI reactive antibody response for the Y2 HA against the Guang/19 strain. Additionally, the COBRA Y2 and Cal/09 HA proteins both share the same 7 potential N-linked glycosylation motifs, NXS/T where X is any amino acids except proline, while Guang/19 has one extra motif in antigenic site Sa at position 179 (22). This additional glycosylation is likely not responsible for differences in the HAI activity between Cal/09 and Y2 as neither HA protein contains the potential glycosylation at site 179 that is present in the Guang/19 sequence. Despite the differences in amino acid sequence between the Y2 and Cal/09 HA, both mRNA vaccines elicited immune responses that inhibited replication of the Cal/09 virus in the lungs following challenge. This is likely due to antibodies that bind to conserved regions in or near the receptor binding site of the virus that are also observed in the HAI assay, but other antibodies directed toward other conserved regions, like the HA stalk, could be playing a role in the observed viral protection (64).

The NG2 H3 mRNA vaccine elicited protective HAI antibody titers against 6 of the last 7 viruses that were selected by the WHO for inclusion in the annual influenza vaccine from 2014-2020, while the Kan/17 mRNA vaccine only generated protective levels of antibodies against H3N2 viruses from 2014-2017. When compared to the Kan/17 H3 mRNA vaccine, the COBRA NG2 H3 HA vaccine also induced significantly higher HAI titers against every H3N2 influenza virus strain, except the matched Kan/17 H3N2 influenza virus (**Figure 4**). This virus belongs to clade 3c.3a, while NG2 HA sequence more closely resembles HA sequences from viruses in clade 3c.2a. These two viral families are typically antigenically distinct and the differences in immunogenicity are likely driven by a N-linked glycosylation at site 160 in antigenic site B (65). Viruses from clade 3c.3a typically lack this glycosylation at site 160, exposing antigenic epitopes which may be covered up in 3c.2a viruses that have this glycosylation (5, 66). The glycosylation at site 160 impacts the HA sialic acid binding specificity, which plays a role in the HA protein binding to host cell receptors (65, 67). NG2 and Kan/17 HA proteins also differ by 11 amino acids and 4 of those residues reside in antigenic sites of the H3 HA protein. In antigenic site A, there are two amino acid differences, Kan/17 possesses a 137N and a 160K, and NG2 has a 137K and 160S. In both locations, the presence of a lysine likely changes the protein structure by introducing a positive charge to the respective molecules (68). Additionally, the 160S in the NG2 amino acid sequence introduces a potential glycosylation site that is not present in the Kan/17 amino acid sequence. Antigenic site B is typically the immunodominant epitope in H3N2 virus HA proteins, and single amino acid substitutions in this region are capable of causing changes in the observed immunogenicity of HA antigens (69). In this antigenic site, the Kan/17 HA amino acid sequence has a 175S, and NG2 has a 175Y. Serine is a polar and hydrophilic amino acid, while tyrosine has a large side chain with polar and non-polar features, but also contains an aromatic ring that could produce structural differences between the Kan/17 and NG2 HA proteins in antigenic site B (63). The final amino acid difference in antigenic sites between Kan/17 and NG2 HA resides in site E, at position 107, where Kan/17 HA contains an asparagine and NG2 has a serine. Asparagine is an amidic amino acid and serine is a hydroxylic amino acid, but both amino acids are polar, hydrophilic, and uncharged at neutral pH (62, 63). Therefore, it is unlikely that the differences in immunogenicity between Kan/17 and NG2 HA are being influenced by the amino acid differences at site 107. The NG2 HA mRNA vaccinated animals had higher HAI and neutralization antibody titers than Kan/17 against the future drifted viral strains from 2019-2020 (**Figures 4**, **5**). The NG2 HA sequence has the same amino acids in sites 107, 137, 160, and 175 as SA/19, HK/19, and Tas/20, which are not shared with the Kan/17 HA sequence. These amino acid differences are likely driving the antigenic differences between Kan/17 and NG2 HA protein against these future drifted H3N2 isolates, but specific site directed mutagenesis experiments at these 4 amino acid positions would need to be conducted to specifically determine which residues are responsible for these observed differences in immunogenicity.

In comparison to the Kan/17 HA mRNA vaccinated animals, the animals that received the NG2 HA mRNA vaccines had significantly lower HAI titers against the Kan/17 virus, however when these animals were challenged with this virus, the NG2 HA mRNA vaccinated animals were able to prevent viral replication in the lungs at day 3 post infection at a similar level as the Kan/17 mRNA vaccine (**Figure 7**). In addition to generating antibody responses directed towards the HA protein head region observed in the HAI assay, COBRA HA vaccination also elicits antibodies that bind to the stem region of the HA protein (70). Therefore, it is possible that antibodies binding to conserved regions of the HA stem are contributing to the prevention of viral replication in the lungs (64). Additionally, the Fc effector function of the antibodies generated through COBRA HA mRNA vaccination are unknown and could be contributing towards the stimulation of a protective cellular immune response that aids in viral clearance. As a result, future studies will investigate the cellular responses that are induced by the Fc regions of antibodies generated through COBRA HA mRNA vaccination.

Much like many other contemporary human H3N2 viruses, the A/Kansas/14/2017 strain used in this study does not cause severe weight loss or mortality in naïve mice (71). However, it was chosen as a challenge virus for this study because unlike other modern H3N2 isolates it does cause moderate weight loss, and replicating virus can be recovered from the lungs of infected mice at day 3 post infection (**Figure 7**). Although influenza viruses can be serially passaged in mice to increase their pathogenicity, we chose to use the human isolate to challenge the animals as mouse adaptation can introduce amino acid substitutions in the HA protein that are rare in circulating human H3N2 viruses (72). Additionally, because most modern H3N2 viruses are not very pathogenic in mice we chose to assess the theoretical protective efficacy of these H3 mRNA vaccine candidates *in-vitro* by examining HAI and neutralizing antibody titers elicited against future drifted H3N2 isolates from 2019-2020. Mice vaccinated with the NG2 mRNA generated higher HAI reactive and neutralizing antibody responses against these isolates than mice that were given the Kan/17 mRNA vaccine.

The bivalent and monovalent formulations of both COBRA and WT mRNA vaccines elicited similar HAI reactive antibody responses that were not statistically different, p>0.05, however, larger differences in antibody responses were observed between the formulations in the neutralization assays (**Figure 5**). This could be due to immunodominance issues that occur when the H1 and H3 HA mRNA vaccines are co-administered. People vaccinated with multivalent influenza virus vaccines have reduced antibody titers against H3N2 HA proteins compared to immune responses elicited to other vaccine components (73, 74). Additionally, competition between vaccine antigens can cause differential immune responses to the individual vaccine components which has occurred in other multivalent vaccine platforms, such as those for dengue fever and human papillomavirus (75). However, vaccines that include multiple antigens in quadrivalent mRNA vaccines demonstrated no difference to monovalent vaccines in NHPs in regards to their elicited humoral immune response (18, 26). Therefore, optimizing the relative doses of the H1 and H3 HA mRNA vaccines to maximize the elicited antibody responses to all the vaccine components equally will be investigated in the future.

Overall, mice that were vaccinated with mRNA-LNP vaccines encoding COBRA H1 and H3 HA proteins had significantly higher protective HAI reactive antibody responses against future drifted viruses from 2019-2020 than animals vaccinated with wild-type H1 and H3 HA mRNA vaccines. Thus, highlighting the potential benefits of incorporating computationally optimized vaccine antigens over antigens from circulating viruses into seasonal influenza vaccines which typically provide less protection against antigenically drifted strains of influenza. In this study this was more apparent for the H3N2 influenza viruses which are more antigenically diverse than the H1N1 viruses isolated since 2009. However, previous iterations of H1 COBRA HA antigens have shown the ability to produce protective antibody titers against antigenically distinct seasonal H1N1 viruses isolated before and after the 2009 H1N1 pandemic (41). Future studies will utilize site directed mutagenesis to determine the specific amino acids that cause differential immune responses between the COBRA and wild-type HA vaccine antigens. Additional follow up studies will also involve animals previously exposed to H1N1 and H3N2 influenza viruses. This will simulate how COBRA mRNA vaccines would perform in a population, like human beings, that have immunological memory to influenza from previous vaccinations and infections (2). These types of studies will also allow the investigation of how memory B and T cell populations are recalled in response to COBRA mRNA vaccination. In this manuscript, mice that were immunologically naïve to influenza required two vaccinations to elicit seroprotective antibody titers. In the context of pre-immunity, the stimulation of memory B and T cells will likely generate immune recall responses and possibly reduce the number of vaccinations required to establish seroprotective antibody titers, and thus will be the focus of future studies. However, the results presented in this manuscript demonstrate the immunological benefits of combining the *in-silico* COBRA HA antigen design technology with an mRNA vaccine delivery platform and highlights the need to continue investigating these next generation vaccines as viable options to improve the efficacy of seasonal influenza vaccines against current and future drifted strains of influenza viruses.

## Data availability statement

The raw data supporting the conclusions of this article will be made available by the authors, without undue reservation.

## Ethics statement

The animal study was approved by University of Georgia, Institutional Animal Care & Use Committee (IACUC). The study was conducted in accordance with the local legislation and institutional requirements.

## Author contributions

JA: Writing – review & editing, Writing – original draft, Visualization, Validation, Supervision, Project administration, Methodology, Investigation, Formal analysis, Data curation, Conceptualization. TR: Writing – review & editing, Supervision, Resources, Funding acquisition, Conceptualization.
